# Dairy sire mating recommendations to limit extremes and target intermediate optima

**DOI:** 10.3168/jdsc.2026-1037

**Published:** 2026-06-11

**Authors:** D.P. Berry

**Affiliations:** Teagasc, Animal & Grassland Research and Innovation Centre, Moorepark, Fermoy, Co. Cork, P61 P302, Ireland

## Abstract

Traditional genetic selection using total merit indexes operates under the simple assumption that higher is better. Some traits, however, are subject to preferred upper or lower thresholds and some may have intermediate optima. Therefore, mating recommender systems should consider these herd-specific breeding goals. Mating recommendations in this study were performed at a herd level for dairy cattle using a constrained optimization approach based on simulated estimates of genetic merit for 9 traits or subindexes. A total of 10,000 herds, each with 200 females, were simulated. The top 100 sires from a simulated population were chosen to represent the pool of candidate sires. Within each herd separately, candidate female–male pairings were evaluated on a mate utility function that combined parental average genetic merit for the 9 traits or subindexes. The utility function penalized matings expected to generate progeny exceeding predefined thresholds (milk yield was used as an example trait) or deviating from an intermediate optimum (cow liveweight was used as an example trait), thereby reflecting both avoidance of genetic extremes and targeting of biologically and system-relevant optima. Imposing a linear penalty on the parental average genetic merit for a trait exceeding a given threshold reduced, considerably, the number of recommended matings that had a parental average genetic merit exceeding that threshold. For the vast majority of herds, no mating that exceeded that parental average genetic merit threshold was recommended but it was unavoidable in some herds because of the presence of females with extreme genetic merit for the trait and the absence of sires capable of delivering the desired parental average genetic merit. Including the threshold penalty had minimal effect on the mean total merit index of the recommended matings. Preselection of candidate dams before mating recommendations, although slightly reducing the proportion of progeny exceeding the threshold, resulted in a lower mean total merit index of the recommended matings. Penalizing matings that generated parental averages deviating from an intermediate optimum resulted in recommended matings that, although lower in genetic merit for the total merit index, were more homogeneous in parental average for the trait in question. Although preselecting dams for mating consideration did not alter the proportion of matings deviating from the optimum, it resulted in a lower mean total merit of the matings. Although the specific conclusions of the present study are dependent on the simulated population, the overarching finding is that developing a utility function that penalizes matings exceeding defined thresholds or deviating from desired optima can support mating recommendations aligned with herd-specific breeding goals that are nonlinear in nature.

## Highlights

• Mating recommender systems can limit extreme genetic merit for progeny traits.
• Mating recommender systems can target intermediate optima for genetic merit.
• Nonlinear breeding goals can be integrated into herd-level mating decisions.

## Graphical Abstract

**Figure:**
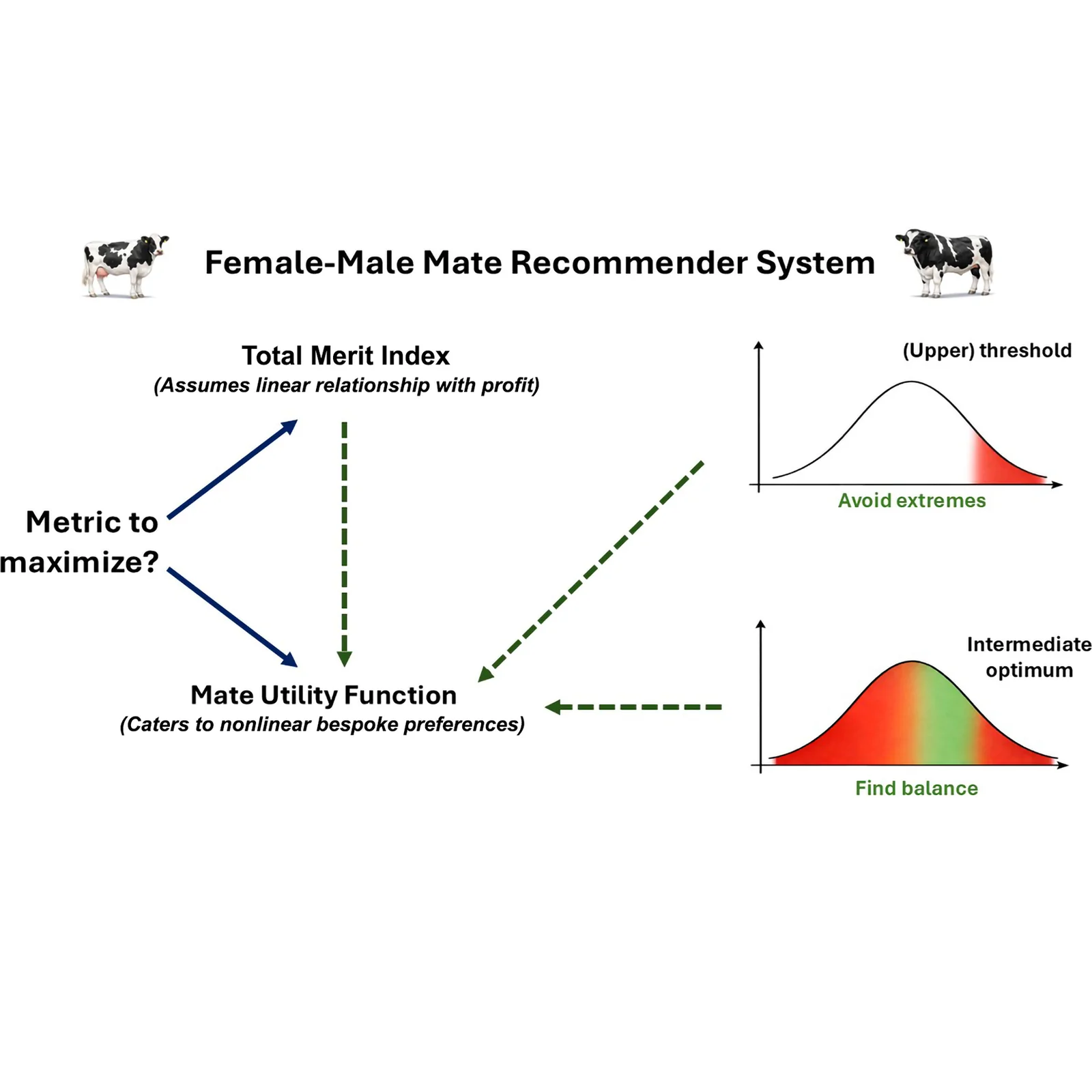
**Summary:** Improved digital support tools are needed to inform on-farm mating decisions with due consideration to the nuances of individual producers or breeders for certain traits or subindexes. These preferences may include avoiding extremes for individual traits or targeting intermediate trait optima. Recommended mating systems that focus solely on maximizing the total merit index of recommended matings (even when accounting for other considerations, such as coancestry) fail to properly consider these. Simulations were used to evaluate the effect of applying thresholds and desired optima to parental average genetic merit for individual traits in a mating recommender system. Matings expected to generate progeny that deviate from these criteria were penalized. The results demonstrate that it is possible to design mating programs to minimize the deviations in expected progeny genetic merit from desired thresholds or intermediate optima. Genetic gain for the overall total merit index suffered only slightly.

The volume and complexity of management decisions on modern dairy farms continue to increase, placing greater demands on producers. Two critical on-farm breeding decisions concern (1) the choice of dairy sires for consideration in the sire team, and (2) the allocation of matings between the selected team of candidate sires and each individual female. Feedback from end-users has highlighted limitations of some mating recommender systems in accommodating bespoke herd breeding goals.

Regulatory frameworks or market-based incentives in some jurisdictions discourage production systems operating outside defined thresholds for certain traits. In addition, producers may prefer intermediate optima for traits like cow liveweight. The global availability of a growing pool of dairy sires has increased the complexity of sire selection, with producers expressing a preference for support tools that facilitate the selection of a smaller, optimal team of sires from a much larger candidate pool. The adoption of sex-sorted semen has further changed on-farm breeding practices, with many farmers now preselecting candidate dams on their total merit index. Whether recommended strategies that prioritize total merit indices for candidate dam selection remain optimal under conditions characterized by individual trait thresholds or intermediate optima warrants further investigation.

The objective of the present study was to extend existing dairy sire mating advice recommender systems by incorporating penalties for parental averages that deviate from defined trait thresholds and intermediate optima. A further objective was to evaluate whether preselection of candidate dams constrains progress toward herd-specific breeding goals in the presence of nonlinearity in their breeding goals. These objectives were investigated using simulation-based analyses.

The simulation approach used in this study consisted of 3 sequential components: (1) simulation of female EBVs for 9 traits, which were then used to calculate the total merit index of each female as well as a female utility function; (2) simulation of sire EBVs for the same traits, along with its total merit index where truncation selection was then applied to retain the top sires; and (3) formation of each possible female–male matings using a progeny utility index with user-defined weights per trait which included upper limit thresholds and intermediate optima for traits. The myriad other considerations that can be incorporated into sire recommender systems, such as the coancestry between mates or their carrier status for lethal genetic mutations, were not taken into account in this study so as to isolate the utility function behavior independent of these selection constraints.

The choice of 9 traits was to reflect the 8 subindexes that comprise the Irish national breeding goal, the Economic Breeding Index (Berry at al. 2022) as well as milk yield; milk yield was chosen because it is used to categorize Irish herds on expected organic nitrogen per cow so therefore many farmers have a preferred upper threshold. For simplicity, all 8 subindexes and milk yield will be referred to as traits in this study. The correlations among all trait EBVs were derived from the trait and subindex values of 4,944 high reliability (>70% reliability) Holstein-Friesian artificial insemination sires born since 1990; a correlation matrix was used so the variances of all subindexes were 1. The correlation matrix used is in Table 1.

**Table 1:** Weights per trait or subindex, as well as the correlation structure assumed among all 9 simulated components as well as with the total merit index

Trait or index	Milk yield	Milk subindex	Fertility subindex	Calving subindex	Beef subindex	Liveweight subindex	Management subindex	Health subindex	Carbon subindex
Weight		48.01	90.82	29.79	25.19	25.74	5.33	10.58	15.43
Milk subindex	0.27								
Fertility subindex	−0.25	0.52							
Calving subindex	0.06	0.60	0.66						
Beef subindex	0.20	−0.18	−0.28	−0.25					
Liveweight subindex	−0.48	−0.18	0.11	0.00	−0.59				
Management subindex	0.06	0.23	0.23	0.21	−0.17	−0.03			
Health subindex	0.17	0.27	0.33	0.32	−0.10	−0.21	0.20		
Carbon subindex	−0.75	−0.05	0.66	0.30	−0.50	0.68	0.08	0.02	
Total merit	−0.16	0.70	0.95	0.79	−0.27	0.13	0.27	0.37	0.57

A mixed-integer linear programming framework was used to optimize mating allocations within each simulated herd separately. For each herd, the input data consisted of all female–male combinations with an associated multitrait mate utility index value based on the combined information from the parental average of the 9 individual traits each weighted with user-defined weights, along with penalties, where appropriate, for any deviation from the defined thresholds or optima. The objective of the optimization was to maximize the average mate utility index values across all recommended matings within each herd.

Candidate dam EBVs were simulated as correlated traits by drawing from a multivariate normal distribution with zero mean and the (co)variance matrix based on the correlations presented in Table 1. Estimated breeding values for a total of 200 candidate dams in 10,000 herds were simulated. The same approach was used to simulate EBVs for 3,000 sires. Truncation selection was applied to retain the top 100 sires ranked on their total merit index. This represented the candidate sire pool, which was common to all herds. The herd mean genetic merit per trait was sampled per herd from a uniform distribution varying from −0.3 to +0.3 SD units. This was added to the individual trait EBV of each candidate dam in that herd so as to create interherd variability in mean genetic merit for all traits.

The utility function per trait *j* for each candidate dam *i* was defined as

*θ_i_* = (*d_j_*, *O_j_*, *T_j_*, *λ_j_*, *κ_j_*),

where $d_{j}\in \left\{+1,-1\right\}$ represented the direction of desirability; *O_j_* was the intermediate optimum value for that trait *j*, if specified; *T_j_* was an upper or lower threshold value for trait *j*, if specified; and λ_i_ and κ_i_ represented the penalty for deviating from the intermediate optimum or threshold value, respectively.

Using the notation just described, the EBV for trait *j* for candidate dam *i* was first transformed so that a positive EBV* was more favorable as

$${EBV}_{ij}^{*}=d_{j}\;EBV_{ij}.$$

If an optimum value for trait *j* was specified (*O_j_*), the contribution of trait *j* to the utility index of dam *i* was defined as

$$s_{ij}=-\lambda_{j}\;\left|EBV_{ij}^{*}-d_{j}O_{j}\right|,$$

where *λ_j_* ≥ 0 was a parameter controlling the penalty severity of deviations from the optimum. This penalty could be of any form but was assumed to be linear in the present study and symmetrical around the optimum value. If no optimum was specified, but a threshold *T_j_* was, then the dam EBV in excess of an upper threshold value (*TU_j_*) or below a lower threshold value (*TL_j_*) was penalized, with the contribution of trait *j* to the utility index of dam *i* being defined as

$$s_{ij}=-\kappa_{j}\left[\max\left(0,\;EBV_{ij}^{*}-TU_{j}\right)+\max\left(0,\;TL_{j}-\;EBV_{ij}^{*}\right)\right],$$

where *κ_j_* ≥ 0 was a parameter controlling the penalty severity of excess relative to the threshold; as with the intermediate optimum, this penalty can be of any form but, in the present study, was assumed linear. If neither an optimum nor a threshold was specified, then the contribution of trait *j* to the utility index of dam *i* was simply defined as

$$s_{ij}=EBV_{ij}^{*}.$$

The overall utility index value of dam *i* was then calculated as a weighted sum of the individual trait contributions:

$$Damutilityvalue_{i}=\overset{9}{\underset{j=1}{\sum }}w_{j}\;s_{ij},$$

where the standardized weight for trait *j* (*w_j_*) was that used in the Irish total merit index (Table 1). Because the weight on milk yield in the Irish total merit index is already captured in the milk subindex, no weight was placed on milk yield genetic merit for the base scenario of no trait threshold or optimum. When implemented, additional considerations can easily be incorporated, such as a penalty on the (expected) coancestry among the pair of mates.

For each of the 10,000 herds simulated, the mating program considered all possible female–male combinations formed from the 100 candidate sires and the 200 candidate dams. The utility function for each female–male combination was calculated using the same formulation as used for ranking candidate dams. The only difference was that dam EBVs in the equations were replaced by the parental average EBVs of the prospective progeny. The mixed-integer linear programming problem was solved using the lpsolve package v 5.6.23 in R (R Core Team, 2022). Mixed-integer linear programming is an optimization method used to find the best possible solution (i.e., allocation of sires to dams so that the overall mate utility of the herd was maximized) to a problem while satisfying a set of constraints (e.g., usage rate per sire). Optimal solutions with mixed-integer linear programming are not always guaranteed when a problem is infeasible or not bounded; examples include if too strict a criterion or criteria were imposed (e.g., very low upper threshold) in combination with a limited number of sires available. In the present study, however, optimal solutions would be found. The mean mate utility index of the herd was to be maximized subject to constraints on the number of bulls used and usage rate per usage. Each herd had to use exactly 10 bulls with a minimum usage per bull of 5% and a maximum usage rate of 20%. Each female could only be mated once.

Only 2 traits were focused on for applying a threshold and intermediate optimum. A desired upper threshold on genetic merit for milk yield at 0.5 SD units above the entire population mean was set for one series of scenarios. For genetic merit for liveweight, 2 different intermediate optima were explored across different scenarios where the penalty for deviations was altered: 0.5 SD or 0.0 SD. For milk yield, different weights on genetic merit for milk yield were explored varying from 0 to 100 with the penalty on genetic merit estimates in excess of the threshold also varying by scenario tested from 1 to 100; the weight and penalty was only applied to parental average genetic merit estimates of milk yield above the threshold. For the liveweight intermediate optimum scenarios, the weighting on liveweight within the utility function was as per the Irish total merit index but the linear penalty applied to deviations from the optimum varied from 1 to 20; the penalty was symmetrical each side of the intermediate optimum.

In a supplementary analysis of each scenario, preselection was also applied to the candidate dam utility index and only those excelling (i.e., top 70%) in the utility index per herd were considered for mating recommendations. Summary statistics for the resulting recommended matings were compared against when all candidate dams were considered for mating but the top 70% of the resulting mating recommendations ranked on the mate utility function were selected.

Summary statistics for a selection of the different scenarios explored are in Table 2. Across the 200 million possible mating combinations (i.e., 200 females per herd × 100 sires × 10,000 herds), 15.7% exceeded a parental average genetic merit threshold of 0.5 SD for milk yield. The percentage of possible matings combinations per herd exceeding the 0.5 SD on parental average genetic merit for milk yield ranged from 7.9% to 25.5%, indicating that all herds contained at least some female–male candidate combinations whose parental average exceeded the milk yield threshold. The mean total merit index across all possible matings was €170.24 (Table 2).

**Table 2:** Mean (SD) total merit index (€), parental average genetic merit for milk yield and liveweight (in SD units), and the percentage of matings and herds exceeding predefined milk yield thresholds or deviating more than 0.5 or 1.0 SD units from the liveweight optimum for all candidate matings, as well as across the different scenarios explored

Item	Candidates	No threshold or optimum			Upper threshold			Intermediate optimum (0.5 SD)		
		All	Top 70% dams	Top 70% matings	All	Top 70% dams	Top 70% matings	All	Top 70% dams	Top 70% matings
Total merit index	170.24 (83.98)	260.47 (99.65)	298.59 (73.48)	311.54 (70.12)	260.47 (96.90)	298.59 (71.44)	309.71 (69.11)	260.24 (86.77)	297.96 (66.96)	303.68 (59.51)
Parental average milk yield	0.14 (0.34)	−0.48 (0.71)	−0.52 (0.70)	−0.65 (0.64)	−0.48 (0.59)	−0.52 (0.61)	−0.60 (0.58)	−0.48 (0.60)	−0.50 (0.60)	−0.57 (0.55)
Parental average liveweight	0.15 (0.71)	0.34 (0.75)	0.37 (0.74)	0.50 (0.66)	0.34 (0.72)	0.37 (0.71)	0.48 (0.64)	0.32 (0.27)	0.33 (0.27)	0.34 (0.26)
Matings > 0.5 SD threshold	15.72%	8.35%	7.30%	3.13%	0.06%	0.04%	0.06%	5.70%	5.26%	2.87%
Matings deviating >0.5 SD from optimum	51.63%	51.34%	51.03%	46.03%	49.47%	49.01%	44.78%	11.42%	10.44%	10.96%
Matings deviating >1 SD from optimum	3.84%	5.68%	5.82%	6.00%	4.96%	5.14%	5.49%	0.0005%	0.0003%	0.0003%
Herds >0.5 SD threshold	100%	100%	100%	100%	11.00%	5.50%	8.44%	100%	99.98%	95.53%
Herds >0.5 SD optimum	100%	100%	100%	99.99%	100%	100%	100%	100%	99.99%	99.99%
Herds >1 SD optimum	100%	100%	99.60%	99.89%	99.98%	99.81%	99.78%	0.09%	0.04%	0.04%

When no upper threshold on parental average milk yield or optimal liveweight was imposed, the mean total merit index of the resulting recommended matings was €260.47 (Table 2). The mean total merit of the top 70% of recommended matings was €311.54. Of the recommended matings, 8.4% exceeded the 0.5 SD threshold in parental average genetic merit for milk yield. When no restrictions were imposed, all herds had some recommended matings that would generate a parental average genetic merit for milk yield of >0.5 SD, with the percentage of recommended matings per herd exceeding this threshold varying from 2% to 18%.

Despite applying a linear penalty to matings exceeding the parental average milk yield threshold of 0.5 SD, 1,153 out of the 200,000 recommended matings still exceeded this parental average milk yield threshold. This number did not change irrespective of either the weight placed on parental average milk yield in the mate utility function or the magnitude of the linear penalty imposed. Nonetheless, this represents a substantial reduction from the 167,050 recommended matings when no threshold was imposed.

Of the recommended matings where an upper threshold on parental average genetic merit for milk yield was imposed, 26 had a parental average genetic merit for milk yield of >1 SD, far above the threshold of 0.5 SD units. On deeper examination, the mean (SD) milk yield genetic merit of the dams of these matings was 4.56 (0.27) SD units; the lowest milk yield genetic merit for the 100 candidate sires considered was −2.96 SD units with only 2 of the candidate sires (both of which were ranked in the lower half on total merit) having a milk yield genetic merit sufficiently negative to deliver a parental average milk yield genetic merit of <1 SD unit. In fact, no candidate sire existed in the simulated dataset to deliver a parental average genetic merit for milk yield below the threshold of 0.5 SD units for these females. This explains why 1,153 recommended matings continued to exceed 0.5 SD on parental average milk yield. The same outcome can undoubtedly happen in practice, particularly when upper thresholds are set at low levels relative to the herd, or lower thresholds are set at high levels relative to the herd. Nonetheless, of the 10,000 herds simulated in the present study, 89.1% had no recommended mating that had a parental average genetic merit for milk yield of >0.5 SD units (Table 2); this is in direct contrast to the unrestricted scenario where all herds had at least 1 such recommended mating.

When retaining just the top 70% of the recommended matings ranked on their mate utility index, the percentage of recommended matings exceeding the 0.5 SD threshold on parental average genetic merit for milk yield declined from 3.13% when no threshold was imposed within the mate utility function to 0.06% where a linear penalty of 1 was imposed (Table 2) and to 0.03% when the linear penalty was increased to 100. If, however, only the top 70% of candidate dams ranked on their utility index were considered for mating, the proportion of recommended matings exceeding 0.5 SD threshold for parental average milk yield decreased even further (Table 2). For example, where no threshold formed part of the candidate dam utility index (i.e., candidate dams selected just on total merit index), the percentage of candidate matings exceeding the 0.5 SD milk yield threshold halved from 15.7% to 7.30% when the top 70% of dams were used as candidate dams (Table 2). When only the top 70% of candidate dams ranked on their utility index with a weight of 1 on milk yield and penalty of 1, the percentage of recommended matings resulting in a parental average genetic merit for milk yield in excess of 0.5 SD was just 0.04%, down from 0.06% if instead based on the top 70% of recommended matings where all dams were considered candidates. Prior selection of candidate dams on their utility index that penalizes excessive genetic merit for traits of interest is therefore potentially one successful strategy to reduce the likelihood of matings being recommended that generate progeny with extreme parental average genetic merit estimates; in such situations, some of the dams with excessive milk yield estimates of genetic merit have already been excluded from consideration.

The mean parental average total merit index of the recommended matings was, however, influenced by the process of selection on either the candidate dams or the resulting parental average estimates. In reality, most herds do not use all the females in the herd to generate replacements. Specifically, differences in the mean expected progeny total merit existed depending on whether the candidate dam pool was restricted to the top 70% ranked on their individual utility index, or whether all dams were retained as candidates and selection was instead based on the top 70% of recommended matings ranked on the mating utility index. Under a scenario in which genetic merit for milk yield was assigned a weight of 1 in the utility function, and a penalty of 1 was applied for exceeding the imposed threshold, restricting the candidate dam pool to the top 70% resulted in a mean parental average total merit index that was 0.13 SD units lower than that obtained when all candidate dams were eligible but only the top 70% of the resulting matings, based on the mating utility index, were selected. This occurred because some high-merit dams exceeding the milk yield threshold were excluded before mating allocation. Therefore, excluding dams solely because they exceed imposed thresholds may reduce overall genetic gain.

In the described scenario, an upper threshold was imposed on milk yield because organic nitrogen excretion per cow in Ireland is linked to herd milk yield. Accordingly, producers may set an upper threshold on herd mean genetic merit for milk yield. More generally, threshold-based penalties may be relevant for traits such as direct calving difficulty, SCC, disease susceptibility traits, or carcass weight or fat score. Results demonstrated that mating recommendations exceeding predefined thresholds could be greatly reduced, and often avoided entirely, although success depended on the availability of sufficiently complementary sires for extreme females.

Two alternative optima for liveweight genetic merit were separately evaluated: a target optimum of 0.5 SD units and a target optimum of 0.0 SD units. Across the 200 million possible candidate matings, 51.63% had a parental average genetic merit for liveweight more than half a SD from the 0.5 SD unit optimum (Table 2), with 49.87% being more than half a SD from the 0.0 optimum; the respective percentages of candidate matings more than 1 SD from the optimum of 0.5 SD units or 0.0 SD units was 3.84% and 17.20%.

When no optimum or threshold constraints were considered in the mate utility function, the mean (SD) parental average genetic merit for liveweight for the recommended matings was 0.34 (0.75) SD units (Table 2). On average, 58.63% and 51.34% of the recommended matings were more than half a SD from 0.0 units and 0.5 units, respectively; the respective percentages more than one SD from 0.0 and 0.5 units was 24.35% and 5.68%. In the scenario of no imposed intermediate optimum (or threshold), all herds had some recommended matings that would generate a parental average genetic merit for liveweight that was more than half a SD from either 0.0 units or 0.5 units; all herds had some recommended matings that would generate a parental average genetic merit for liveweight that was more than 1 SD from 0.0 units, and all but 1 herd out of the 10,000 herds simulated had at least 1 recommended mating that would generate a parental average genetic merit for liveweight of more than 1 SD from 0.5 units.

When the optimum parental average genetic merit for liveweight was set at 0.5 SD units, the mean (SD) parental average liveweight genetic merit was 0.32 (0.27) units where a linear penalty of 1 was imposed on deviations from this optimum; the respective mean (SD) was 0.43 (0.22) units when a linear penalty of 10 per unit SD was imposed. In a supplementary analysis, an exponential penalty was applied to deviations from the intermediate optimum; in this case the mean (SD) parental average liveweight genetic merit was 0.34 (0.24) units, where a penalty of 1 was applied, although increasing the penalty to 10 did not affect the outcome. This means that relative to when no penalty was applied, the variability in parental average genetic merit for liveweight decreased considerably, and applying a strong penalty also resulted in a shifting of the mean of the parental average for liveweight toward the optimum desired (here 0.5 SD units). When the optimum parental average liveweight genetic merit was set at 0.0 units, the mean (SD) parental average for liveweight was 0.07 (0.29) with a linear penalty of 1, and it was 0.01 (0.15) units when a linear penalty of 10 was applied. The mean parental average total merit index of the recommended matings where 0.5 SD units of parental average genetic merit for liveweight was the optimum and a penalty of 1 or 10 was used was €260.24 (Table 2) and €254.77, respectively; when the optimum parental average genetic merit for liveweight was 0.0 units, the mean (SD) total merit index was €254.30 (€84.96) and €238.96 (€85.53) when a penalty of 1 or 10 was used, respectively. Therefore, for the higher liveweight optimum a negligible effect occurred on the mean total merit of the recommended matings relative to when no restriction was imposed, but the variability in total merit among the recommended matings was lower when the mate utility function considered an intermediate optimum. It should be noted that only a weak correlation of 0.13 existed between the liveweight trait and the total merit index (Table 1) and that the 0.5 SD unit optimum was not extreme.

Per herd, when the desire was to have an optimum parental average genetic merit for liveweight of 0.5 SD units, more than 99% of the herds had no recommended mating that would generate a parental average liveweight genetic merit more than 1 SD from this optimum (Table 2); this compares to just 1 herd out of the 10,000 herds fulfilling this criterion when no optimum was considered in the mate utility function. When the desired optimum for parental average liveweight was set at 0.0 units, then 84% and 95% of herds had no recommended mating that would generate a parental average liveweight genetic merit more than 1 SD from 0.0 SD where the linear penalty was 1 and 10, respectively. Preselecting the top 70% dams for consideration for mating based on their utility index did not affect the percentage of matings or, indeed, the percentage of herds with at least 1 recommended mating that was >0.5 SD from the threshold of 0.5 SD units (Table 2); this strategy did, however, result in a lower mean total merit index of the progeny by 0.13 SD units relative to all dams being considered and instead the top 70% of those recommended matings instead chosen (Table 2).

Preference for intermediate phenotypes is common in dairy systems, particularly for traits such as animal size, gestation length, and milking speed. Results from the present study demonstrated that mating recommendations could both shift the mean and reduce variability in parental average genetic merit. Several considerations must be taken into account, however, when pursuing greater uniformity among animals based on mate selection. First, Mendelian sampling during gametogenesis ensures that genetic variability in progeny will exist around their midparent mean. Second, although cow liveweight is heritable, the within-breed narrow-sense heritability is approximately 0.61 (Berry and McCarthy, 2021), indicating that nongenetic factors will also contribute substantially to observed phenotypic variation in traits such as liveweight. Assuming a genetic SD for cow liveweight of 35.4 kg and a heritability of 0.61 (Berry and McCarthy, 2021), the expected phenotypic SD among progeny with the same parental average genetic merit for liveweight from unrelated parents of the same breed, both with an estimated reliability of 50% for genetic merit for liveweight, is 37.8 kg. This corresponds to an expected difference in mature cow liveweight of 133 kg between the heaviest and lightest 10% of contemporary progeny; this increases only to 146 kg when the SD in parental average genetic merit for liveweight is 1 unit. Consequently, achieving phenotypic uniformity through mating alone in an outbred population of the same breed is challenging, even for a moderate-to-highly heritable trait. One potential strategy to reduce herd variability is to generate surplus progeny, genotype, and select based on their genomic evaluations, thereby capturing the Mendelian sampling effects. Nevertheless, it is important to recognize that efforts to increase uniformity inevitably incur a (small) loss in potential genetic gain depending on the targets.

Conclusions drawn on the effect of the 2 main strategies imposed in the present study are a function of how the data were simulated, including, importantly, the covariance components among the 9 traits and total merit index, their relative weighting, the traits where the constraints were imposed, the stringency of the thresholds, and the imposed penalty on deviations from these thresholds. These will certainly differ between populations. Although relatively strong similarities exist in the suites of traits included in dairy cow breeding objective globally (Cole and VanRaden, 2018), the correlations among breeding objectives are not perfect and differences are largely the result of differences in covariances among traits (Berry et al., 2026). A mating recommender system will also consider other mate features of interest such as a penalty on coancestry among the parents (Carthy et al., 2019), as well as whether the parents are both carriers of the same lethal genetic mutation (Bengtsson et al., 2022). Including these complications into the process of sire mating advice can make the mating decisions for a herd unwieldy, necessitating a linear programming (or alternative) strategy to solve.
